# A clinical drug–drug interaction study to evaluate the effect of a proton-pump inhibitor, a combined P-glycoprotein/cytochrome 450 enzyme (CYP)3A4 inhibitor, and a CYP2C9 inhibitor on the pharmacokinetics of vismodegib

**DOI:** 10.1007/s00280-016-3020-z

**Published:** 2016-05-06

**Authors:** Vikram Malhi, Dawn Colburn, Sarah J. Williams, Cornelis E. C. A. Hop, Mark J. Dresser, Priya Chandra, Richard A. Graham

**Affiliations:** Genentech, Inc., 1 DNA Way, South San Francisco, CA 94080 USA; F. Hoffmann-La Roche, Ltd., Welwyn Garden City, UK; Denali Therapeutics, South San Francisco, CA USA; Theravance Biopharma, South San Francisco, CA USA

**Keywords:** Pharmacokinetic, Clinical drug–drug interaction, Clinical pharmacology, Proton-pump inhibitor, CYP inhibitors, P-glycoprotein inhibitor

## Abstract

**Purpose:**

The Hedgehog pathway inhibitor vismodegib exhibits pH-dependent solubility, and in vitro studies have shown that vismodegib is a substrate of P-glycoprotein (P-gp) and is metabolized by cytochrome P450 (CYP) 2C9 and 3A4. The objective of this four-arm parallel study in healthy subjects was to evaluate the effect of the proton-pump inhibitor rabeprazole, the P-gp/CYP3A4 inhibitor itraconazole, and the CYP2C9 and 3A4 inhibitor fluconazole on vismodegib steady-state pharmacokinetics.

**Methods:**

Cohorts included a control arm (*n* = 22), in which vismodegib 150 mg was administered once daily (QD) for 7 days, and 3 arms in which vismodegib was co-administered QD for 7 days with rabeprazole 20 mg (including a 4-day lead-in; *n* = 24); itraconazole 200 mg (*n* = 22); or fluconazole 400 mg (*n* = 22).

**Results:**

Area under the vismodegib concentration–time curve from zero to 24 h (AUC_0–24h_) at steady state was lower with concomitant rabeprazole administration relative to vismodegib alone [geometric mean ratio (GMR), 86.2 (associated 90 % confidence interval [CI], 76.1, 97.7)]. There was no effect of itraconazole on steady-state exposure of vismodegib [GMR, 96.4 (90 % CI 84.9, 109.6)]. Co-administration with fluconazole increased vismodegib steady-state AUC_0–24h_ [GMR, 130.9 (90 % CI 115.2, 148.7)]. Co-administration of rabeprazole, itraconazole, and fluconazole had similar effects on the exposure of unbound vismodegib and total vismodegib.

**Conclusion:**

The results of this study suggest that vismodegib can be administered with acid-reducing agents and P-gp and CYP inhibitors without the risk of a clinically meaningful pharmacokinetic drug–drug interaction.

**ClinicalTrials.gov Identifier:**

NCT01772290.

**Electronic supplementary material:**

The online version of this article (doi:10.1007/s00280-016-3020-z) contains supplementary material, which is available to authorized users.

## Introduction

The Hedgehog signaling pathway is a key driver in the pathogenesis of basal cell carcinoma (BCC). Vismodegib (Erivedge^**®**^), a first-in-class oral Hedgehog pathway inhibitor administered once daily (QD), has demonstrated efficacy in patients with advanced BCC [1] and is approved worldwide for the treatment of locally advanced and metastatic BCC.

Vismodegib demonstrates a unique single-dose pharmacokinetic (PK) profile, with sustained plasma concentration over several days contributing to a terminal elimination half-life of 12 days [2]; however, steady-state concentrations (*C*_ss_) are typically achieved within 7 days [3]. Furthermore, vismodegib displays nonlinear PKs with respect to dose, with no increase in steady-state plasma concentrations when increasing the daily dose from 150 to 270 or 540 mg [4]. These unusual PK properties have been attributed to two distinct processes: (1) solubility-limited absorption related to the poor and pH-dependent solubility of vismodegib and (2) high-affinity saturable plasma protein binding. In the single- and multiple-dose absolute bioavailability vismodegib PK study in healthy subjects, it was observed that the oral steady-state exposure or area under the plasma concentration–time curve from zero to 24 h (AUC_0–24h_) after multiple dosing was approximately eightfold lower when compared with the area under the plasma concentration–time curve from zero to infinity after a single dose [3]. However, the fact that the total clearance increased only by approximately twofold (i.e., 81 %) suggests that the bioavailability (i.e., fraction absorbed as first-pass clearance) is low for vismodegib and decreased considerably with multiple dosing. Moreover, in vitro vismodegib binds to both human serum albumin and human alpha-1-acid glycoprotein (AAG), with a higher affinity for AAG [5]. Vismodegib *C*_ss_ and AAG plasma concentration are strongly correlated, with AAG levels explaining most (>70 %) of the PK variability observed in patients [6]. Lastly, vismodegib exhibits slow intrinsic clearance via metabolism in the liver, and the majority of the drug is excreted unchanged in the feces, with most of it being unabsorbed material [7].

Vismodegib is a Biopharmaceutics Classification System Class II molecule and exhibits pH-dependent solubility. The solubility of vismodegib decreases by 10,000-fold from pH 2 to 7, and it could be anticipated that vismodegib PKs might be altered when concomitantly administered with drugs that elevate gastric pH such as acid-reducing agents (ARAs). In addition, vismodegib was identified as a P-glycoprotein (P-gp) substrate on the basis of the results of an in vitro study in Madin–Darby canine kidney cells overexpressing P-gp when transfected with the human *MDR1*. Finally, vismodegib elimination involves multiple pathways, with the major metabolites being formed by cytochrome P450 (CYP) enzymes 2C9 and 3A4 [7].

PK drug–drug interactions (DDIs) can lead to alterations in the efficacy (increased or decreased) and/or safety profile. Consequently, it is essential to assess new agents for interactions with a series of drugs that are likely to be co-administered. The objective of this study was to evaluate the DDI potential when vismodegib is co-administered with an ARA (e.g., proton-pump inhibitors [PPIs] such as rabeprazole, H_2_-receptor antagonists, or antacids) or drugs that may potentially cause a transport or metabolism-based DDI (e.g., P-gp or CYP inhibitors such as itraconazole and fluconazole). Although the risk of a clinically significant impact due to a DDI was predicted to be low based on the available preclinical and clinical data, a clinical study was performed to confirm this. This study was required by the US Food and Drug Administration (who requested an evaluation of vismodegib with ARAs) and the European Medicines Agency (who requested an evaluation of vismodegib with combined CYP2C9 and CYP3A4/P-gp inhibitors).

## Materials and methods

### Study design

This was a phase Ib, open-label, four-arm, parallel design, multicenter, multiple-dose study in healthy female subjects of non-childbearing potential. Subjects were randomly assigned to 1 of 4 treatment arms in a 1:1:1:1 ratio with stratification according to AAG concentration [low (0.5–0.73 g/L), medium (0.74–0.96 g/L), or high (0.97–1.2 g/L)]. Arm A received oral vismodegib 150 mg QD on days 1–7; Arm B received oral rabeprazole 20 mg QD on days 1–4, followed by rabeprazole 20 mg administered 2 h prior to dosing with vismodegib 150 mg QD on days 5–11; Arm C received oral itraconazole 200 mg QD administered 2 h prior to dosing with vismodegib 150 mg QD on days 1–7; and Arm D received fluconazole 400 mg (two 200-mg tablets) QD administered 2 h prior to dosing with vismodegib 150 mg QD on days 1–7.

Subjects were admitted to the clinical research unit the day preceding the first day of study drug administration (day^−1^) and were confined until study completion (day 8 or 12). Study treatments were administered in the morning with approximately 240 mL of water after an overnight fast (not including water) of at least 8 h. During the study, administration of any concomitant medications was prohibited without prior approval of the investigator (other than thyroid hormone replacement therapy with a stable dose for at least 3 months). Additionally, prior to check-in, subjects were to refrain from use of any investigational drug or biologic agent (5 half-lives or 30 days, whichever was longer), H_2_-receptor antagonists (1 month), or antacids (1 month); any medications/products or vaccines, including seasonal flu and H1N1 vaccines (7 days), monoamine oxidase inhibitors, thioridazine, pimozide, or antidepressants (1 month, or 2 weeks for antibiotics), narcotics for pain (2 weeks); or any over-the-counter, nonprescription medications, including vitamins, minerals, phytotherapeutic/herbal/plant-derived preparations, tryptophans, St. John’s wort (7 days or 5 half-lives, whichever was longer), and PPIs (6 months). During the study and for 72 h prior to check-in, subjects abstained from consuming alcohol-, grapefruit-, or caffeine-containing foods or beverages, and they received a standardized diet at scheduled times while confined to the clinical research unit.

### Ethical approval

The study protocol was approved by an independent Institutional Review Board, and the study was conducted in accordance with the provisions of the Declaration of Helsinki and Good Clinical Practice guidelines. Informed consent was obtained from all individual participants included in the study. This trial is registered with ClinicalTrials.gov, identifier NCT01772290.

### Subjects

Eligible subjects were females of non-childbearing potential (postmenopausal for at least 1 year or surgically sterile), aged 18–70 years with a body mass index of 18.0–35.0 kg/m^2^ and in good health based on medical history, physical examination, 12-lead electrocardiogram, and laboratory test results. The acceptable range for serum AAG levels was 0.5–1.2 g/L within 9 days prior to the first dose. Subjects were excluded if they had significant medical history or clinical manifestation of any metabolic, allergic, dermatologic, hepatic, renal, hematologic, pulmonary, cardiovascular, endocrine, gastrointestinal, urologic, neurologic, or psychiatric disorder (as determined by the investigator), Zollinger–Ellison syndrome, Barrett’s esophagus, or cancer.

### PK assessments and analysis

Blood samples were collected for PK analysis of vismodegib prior to dosing and at 0.5, 1, 2, 4, 6, 8, 12, and 24 h postdose on days 1 and 7 (or days 5 and 11 in the vismodegib + rabeprazole arm), and within 2 h prior to dosing on days 2–6 (days 6–10 in the vismodegib + rabeprazole arm). The primary PK parameters of interest were the average plasma concentration (*C*_ss_,_ave_) of vismodegib and the AUC_0–24h_ at steady state on day 7 (or day 11 in the vismodegib + rabeprazole arm). Exploratory PK parameters included vismodegib maximum plasma concentration (*C*_max_), AUC_0–24h_, and time to maximum concentration after a single dose on day 1 (or day 5 for the vismodegib + rabeprazole arm), time to steady state (*t*_ss_; defined as the earlier of 2 consecutive days on which the difference in vismodegib concentrations was <20 %) in the vismodegib and vismodegib + rabeprazole arms only, and the average unbound plasma concentration at steady state (*C*_ss_,_ave_,_u_) using values from 2, 6, and 12 h postdose on day 7 (or day 11 in the vismodegib + rabeprazole arm). PK parameters were calculated using noncompartmental methods with WinNonlin (Pharsight Corporation, Mountain View, CA; Version 5.2); the PK analysis used actual times as recorded on the case report form.

Total vismodegib plasma concentrations were determined by Covance Laboratories Inc.’s Bioanalytical Department (Madison, WI) using a validated high-performance liquid chromatography with tandem mass spectrometric detection (HPLC–MS/MS) analytical procedure [2]. Unbound plasma concentrations of vismodegib were determined by Covance Laboratories Inc.’s Bioanalytical Department (Madison, WI) using a validated HPLC–MS/MS analytical procedure following equilibrium dialysis performed by QPS (Newark, DE) [8].

### Safety assessments

Safety and tolerability of vismodegib were assessed through collection of adverse events (AEs) (incidence, nature, and severity), and also changes in clinical laboratory results, vital signs, electrocardiograms, and physical examination findings.

### Statistical analysis

A total of 96 subjects were planned for enrollment in order to provide at least 20 subjects with evaluable PK results in each of the four treatment arms. This sample size was estimated to be sufficient for the point estimate of the geometric mean ratio (GMR) in AUC_0–24h_ so that between two arms, results would fall within 80 and 125 % of the true value with 90 % confidence, assuming a true ratio of 1, based on the standard deviation of the natural log-transformed AUC_0–24h_ of 0.21 obtained from prior PK studies with vismodegib [9].

To evaluate the relative effect of the interacting drugs on the PK of vismodegib, a statistical model was built to compare the parameters *C*_ss_ and AUC_0–24_ post-day 7 (day 11 for the vismodegib + rabeprazole arm) dose between the control arm (Arm A) and the other study arms: rabeprazole (Arm B), itraconazole (Arm C), or fluconazole (Arm D). The values of *C*_ss_ and AUC_0–24_ were natural log-transformed prior to statistical analysis. The statistical model had treatment arm as fixed effect with four levels (A, B, C, and D) as follows: log (PK parameter) = treatment + random error.

The GMR was estimated for the *C*_ss_ and AUC_0–24_ between Arms A and B, Arms A and C, and Arms A and D. A 90 % confidence interval (CI) was calculated for each GMR of *C*_ss_ and AUC_0–24_ from the statistical model. All other parameters were summarized as descriptive statistics.

Steady state was defined as the earlier of 2 consecutive days (the predose time points) at which the difference in vismodegib concentrations was <20 %. To evaluate the relative effect of rabeprazole (Arm B) on the time-to-reach steady state for daily dosing of vismodegib, *t*_ss_ post-day 1 (day 5 for the vismodegib + rabeprazole arm) was calculated for the control arm (Arm A) and descriptive statistics of *t*_ss_ were provided for Arms A and B.

The PK population included all subjects who received all seven doses of vismodegib and had evaluable PK data. The safety population comprised all subjects who received at least one dose of vismodegib.

## Results

### Demographics and baseline characteristics

A total of 92 subjects were enrolled in the study. All were evaluable for safety, while 90 were evaluable for PK analysis (2 subjects from the vismodegib + fluconazole arm who withdrew from the study on days 3 and 8, respectively, were excluded from the PK analysis). Baseline demographics were well balanced across the four treatment arms (Supplemental Table 1). Excluding the two subjects who withdrew from the study, all subjects were compliant with treatment and received all planned doses of study medication.

### Pharmacokinetic results

#### Effects of rabeprazole on vismodegib PK

**Fig. 1:**
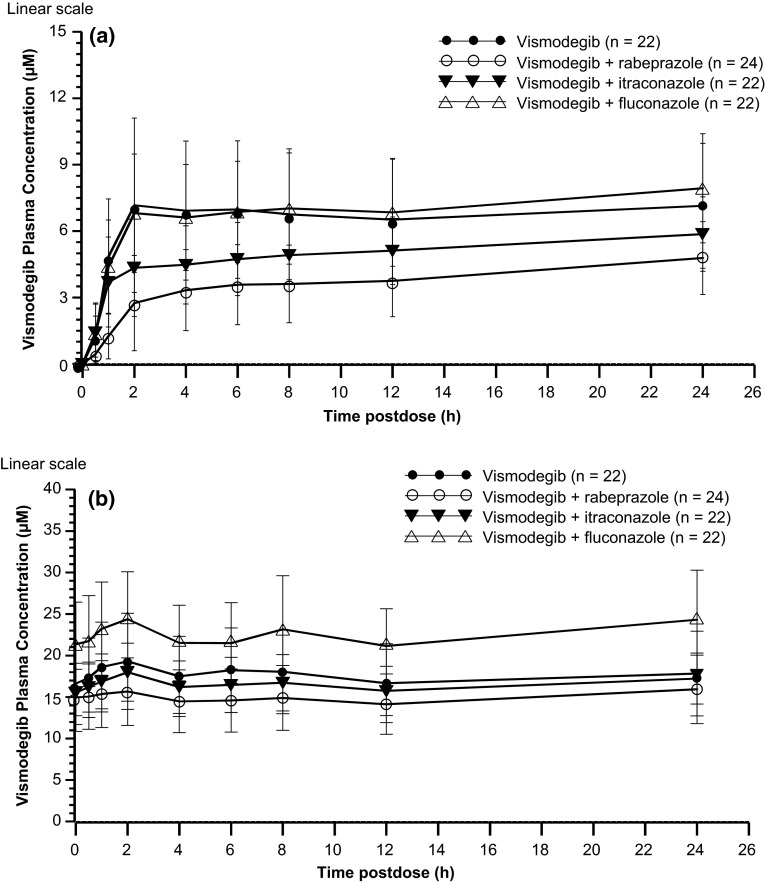
Mean (± SD) plasma concentration–time profiles of vismodegib after **a** single-dose administration of vismodegib (150 mg) and **b** multiple-dose administration of vismodegib (150 mg). *SD* standard deviation

**Fig. 2:**
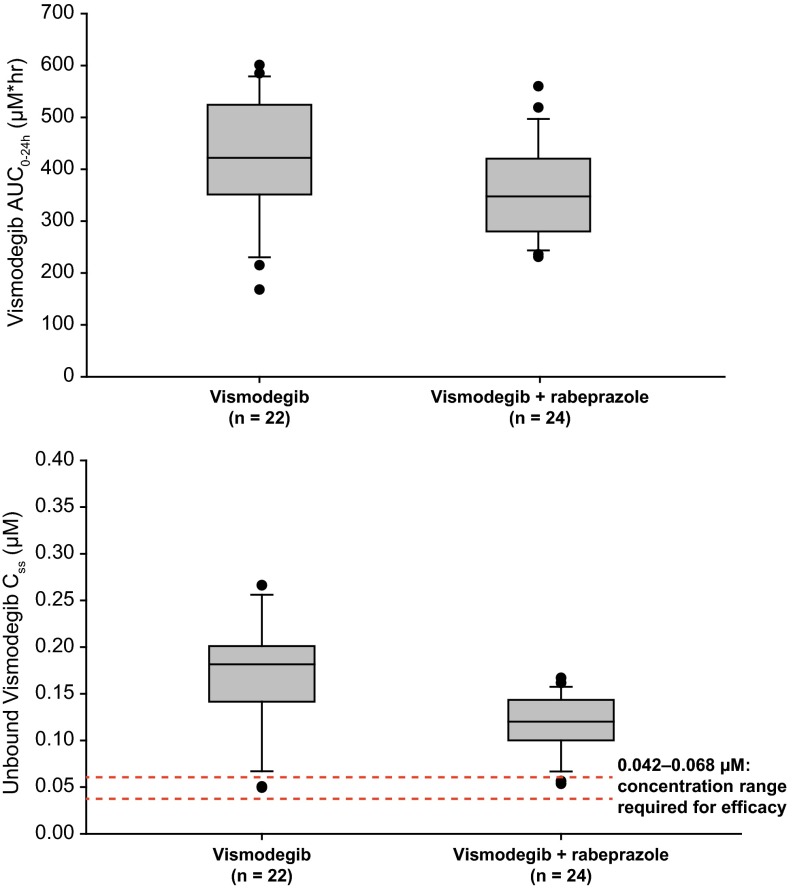
Vismodegib (150 mg) with and without rabeprazole co-administration. *Top*, box plot for vismodegib AUC_0–24h_. *Bottom*, box plot for vismodegib *C*_ss_,_ave_,_u_

Co-administration of rabeprazole was associated with a moderate decrease in single-dose vismodegib exposure compared with vismodegib alone, with a 42 % reduction in geometric mean AUC_0–24h_ (80.7 versus 140 µmol h/L) and a 35 % reduction in *C*_max_ (4.56 vs. 7.00 µmol/L; Table 1; Fig. 1a). Steady-state exposure of vismodegib was slightly decreased by co-administration of rabeprazole, with a 14 % reduction in the geometric mean AUC_0–24h_ and *C*_ss_,_ave_ values compared with vismodegib alone (Tables 1, 2; Fig. 1b). Co-administration of rabeprazole had no effect on the median *t*_ss_, which was 95.9 h in both the vismodegib and vismodegib + rabeprazole arms (Table 1). Vismodegib *C*_ss_,_ave_,_u_ was 33 % lower in the vismodegib + rabeprazole arm compared with the vismodegib arm (Table 1; Fig. 2).

**Table 1:** PK parameters (CV %) for vismodegib following single-dose (day 1 or 5) and multiple-dose (day 7 or 11, steady state) administration of oral vismodegib (150 mg QD)

	Arm A Vismodegib (*n* = 22)	Arm B Vismodegib + rabeprazole (*n* = 24)	Arm C Vismodegib + itraconazole (*n* = 22)	Arm D Vismodegib + fluconazole (*n* = 22)
*Single-dose*				
Collection day	1	5	1	1
AUC_0–24h_ (µmol h/L)	140 (60.0)	80.7 (45.6)	111 (44.3)	159 (35.2)
*C*_max_ (µmol/L)	7.00 (55.8)	4.56 (36.9)	5.65 (37.4)	8.01 (33.2)
*t*_max_^a^ (h)	6.00 (2.0, 24.0)	23.9 (2.0, 24.0)	23.9 (2.0, 23.9)	23.9 (1.0, 24.0)
*t*_ss_^a,b^ (h)	95.9 (48.0, 120.0)	95.9 (71.9, 144.0)	NC	NC
*Multiple-dose*				
Collection day	7	11	7	7
AUC_0–24h_ (µmol h/L)	402 (33.3)	346 (25.5)	388 (20.8)	526 (22.4)
*C*_ss_,_ave_ (µmol/L)	16.7 (33.4)	14.4 (25.4)	16.2 (20.8)	21.9 (22.4)
*C*_ss_,_ave_,_u_ (µmol/L)	0.150 (50.2)	0.101 (32.8)	0.147 (39.9)	0.236 (29.7)

Geometric mean (CV %) data are presented unless indicated

*CV* *%* coefficient of variation, *NC* not calculated, *PK* pharmacokinetic, *QD* once daily

^a^Median (min, max) presented for *t*_max_ and *t*_ss_

^b^ *t*_ss_ estimated after continuous QD dosing (AUC_0–24h_, *C*_max_, and *t*_max_ estimated after the first dose)

**Table 2:** Statistical analysis of between-group differences in vismodegib exposure at steady state

	Treatment	*n*	Geometric LS means^a^	Ratio of geometric LS means to Arm A (%)^b^	90 % CI for the ratio (%)^c^	
					Lower	Upper
AUC_0–24h_ (µmol h/L)	Arm A (vismodegib)	22	402			
	Arm B (vismodegib + rabeprazole)	24	346	86.2	76.1	97.7
	Arm C (vismodegib + itraconazole)	22	388	96.4	84.9	109.6
	Arm D (vismodegib + fluconazole)	22	526	130.9	115.2	148.7
*C*_ss_,_ave_ (µmol/L)	Arm A (vismodegib)	22	16.7			
	Arm B (vismodegib + rabeprazole)	24	14.4	86.3	76.2	97.8
	Arm C (vismodegib + itraconazole)	22	16.2	96.5	85.0	109.7
	Arm D (vismodegib + fluconazole)	22	21.9	130.9	115.2	148.7

*ANOVA* analysis of variance, *CI* confidence interval, *LS* least squares

^a^Geometric LS means from ANOVA, calculated by transforming the natural log means back to the linear scale

^b^Ratio of geometric LS means back-transformed to the linear scale from the difference calculated on the natural log scale (expressed as a percent)

^c^90 % CI for ratio of parameter LS means of natural log-transformed parameter (expressed as a percent). Natural log-transformed confidence limits transformed back to the linear scale

#### Effects of itraconazole on vismodegib PK

**Fig. 3:**
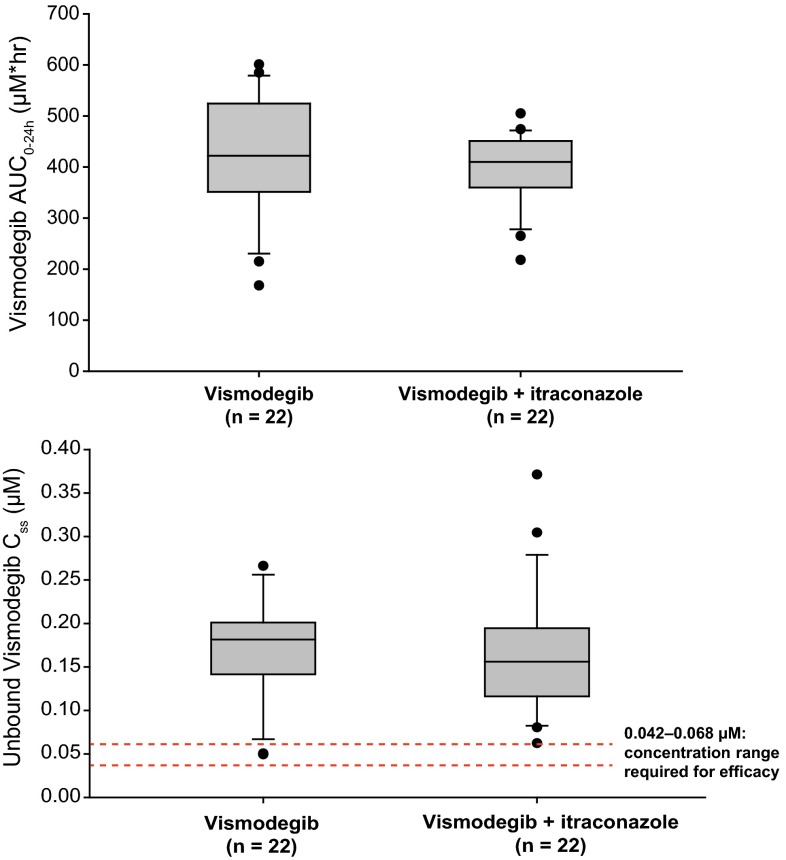
Vismodegib (150 mg) with and without itraconazole co-administration. *Top*, box plot for vismodegib AUC_0–24h_. *Bottom*, box plot for vismodegib *C*_ss_,_ave_,_u_

Compared with vismodegib alone, co-administration of itraconazole decreased the geometric mean single-dose vismodegib AUC_0–24h_ and *C*_max_ by 21 % (111 versus 140 µmol h/L) and 19 % (5.65 vs 7.00 µmol/L), respectively (Table 1; Fig. 1a). Co-administration of itraconazole with vismodegib did not appear to have an effect on steady-state exposure to vismodegib, with similar AUC_0–24h_ and *C*_ss_,_ave_ values observed in the vismodegib and vismodegib + itraconazole arms (Tables 1, 2; Fig. 1b). The 90 % CI for the GMR for AUC_0–24h_ was 84.9–109.6 and for *C*_ss_,_ave_ was 85.0–109.7, suggesting bioequivalence (Table 2). Vismodegib *C*_ss_,_ave_,_u_ was similar in the vismodegib and vismodegib + itraconazole arms (Table 1; Fig. 3).

#### Effects of fluconazole on vismodegib PK

**Fig. 4:**
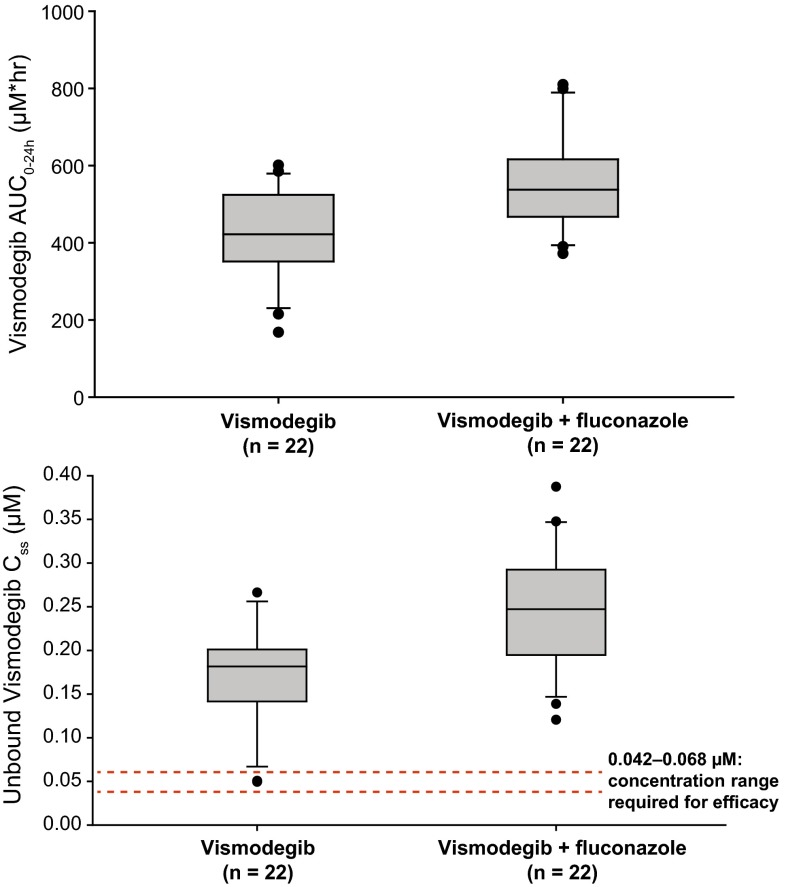
Vismodegib (150 mg) with and without fluconazole co-administration. *Top*, box plot for vismodegib AUC_0–24h_. *Bottom*, box plot for vismodegib *C*_ss_,_ave_,_u_

Following a single dose, co-administration of fluconazole with vismodegib resulted in a 14 % increase in AUC_0–24h_ (159 versus 140 µmol h/L) and *C*_max_ (8.01 versus 7.00 µmol/L) compared with vismodegib alone (Table 1; Fig. 1a). Co-administration of fluconazole with vismodegib resulted in a moderate increase in vismodegib exposure at steady state, with AUC_0–24h_ and *C*_ss_,_ave_ geometric mean values approximately 31 % higher in the vismodegib + fluconazole arm compared with the vismodegib arm (Table 1; Fig. 1b). Vismodegib *C*_ss_,_ave_,_u_ was 1.57-fold higher in the vismodegib + fluconazole arm than in the vismodegib arm (Table 1; Fig. 4), indicating a weak DDI between vismodegib and fluconazole.

Additionally, all subjects enrolled in the study were genotyped to identify genetic polymorphisms of CYP2C9 to further elucidate the effect of this enzyme on the PK of vismodegib. However, it was not possible to fully evaluate any differences in the steady-state PK of vismodegib based on the CYP2C9 genotype, since there were no poor metabolizers enrolled in this study.

### Safety

Overall, 127 treatment-emergent AEs were observed in 49 (53.3 %) subjects across all treatment arms (Supplemental Table 2). The most frequent AEs were headache (13.0 %), constipation (12.0 %), nausea (9.8 %), and diarrhea (8.7 %). All AEs were mild in severity and resolved after study completion. No serious AEs or deaths occurred. The incidence of treatment-emergent AEs was highest with co-administration of rabeprazole and vismodegib (66.7 % of subjects), followed by co-administration of itraconazole and vismodegib (63.6 %), co-administration of fluconazole and vismodegib (41.7 %), and administration of vismodegib alone (40.9 %). Of the 127 treatment-emergent AEs, 92 were considered related to vismodegib. Among subjects receiving rabeprazole, itraconazole, or fluconazole in combination with vismodegib, treatment-emergent AEs were considered related to vismodegib in 33.3, 54.5, and 37.5 % of subjects, respectively. The most frequent AEs related to vismodegib were headache (10.5 %), constipation (10.5 %), nausea (9.3 %), and diarrhea (8.0 %).

## Discussion

The primary goal of this study was to assess potential DDIs between vismodegib and the potent PPI rabeprazole, the strong P-gp/CYP3A4 inhibitor itraconazole, and the moderate CYP2C9 and 3A4 inhibitor fluconazole. PPIs are commonly used for gastroesophageal reflux disease, with clinical benefit attributed to the potent reduction of gastric acid secretion via blockade of the H +/K + ATPase on the gastric parietal cell. Moreover, patients often continue therapy for extended durations without a defined end point [10]. ARAs such as PPIs may alter the solubility of co-administered drugs if the co-administered drug is less soluble at a higher pH, resulting in reduced bioavailability and overall exposure. For example, co-administration of the PPI lansoprazole with atazanavir led to a large reduction in atazanavir bioavailability [11]. Therefore, the atazanavir label recommends the avoidance or a dose reduction of PPIs. Similarly, ARAs have been shown to impair ketoconazole absorption by more than 90 % [12]. Lastly, it is important to note that gastroesophageal reflux disease is common in patients with cancer, and the prevalence of ARA use in this population ranges from 18 to 50 % [13].

Vismodegib is a Biopharmaceutics Classification System Class II molecule and exhibits pH-dependent solubility. As such, the solubility of vismodegib decreases by 10,000-fold from pH 2 to 7. However, the PK effect of co-administering vismodegib with agents that increase gastric pH was previously unknown. In this study, co-administration of the potent PPI rabeprazole with vismodegib resulted in a small reduction in vismodegib on day 1 (42 % reduction in AUC_0–24h_) and an even smaller reduction in steady-state exposure of approximately 14 % in AUC_0–24h_ and *C*_ss_,_ave_ values compared with administration of vismodegib alone. Although unbound vismodegib plasma concentrations were 33 % lower following co-administration with rabeprazole, the results are not clinically meaningful, since the mean unbound concentrations for all subjects were above the predicted preclinical efficacious levels of vismodegib of 0.042–0.068 µM [14]. While *C*_ss_,_ave_,_u_ exhibited a larger change in the presence of rabeprazole compared with *C*_ss_,_ave_, it should be noted there is extensive overlap between the range of the unbound and total vismodegib *C*_ss_ in the presence of rabeprazole compared with the control arm (Fig. 2).

This result was not surprising because there was a low risk for an interaction between vismodegib and a PPI, as the influence of AAG C_ss_ on vismodegib PK has been established, with AAG levels explaining >70 % of the PK variability [data on file]. Furthermore, it was previously observed that extrinsic factors that affect the absorption process such as food and formulation (a function of particle size) had no effect on both unbound and total vismodegib steady-state levels [4, 15]. When vismodegib exposure was evaluated with food, it was observed that a high-fat meal increased single-dose vismodegib exposure relative to the fasted state (up to 38 %), with no corresponding change in *C*_ss_. Vismodegib absorption and hence exposure was increased by food (under single-dose conditions when AAG binding is not saturated); however, with continuous dosing, steady-state vismodegib exposure is instead related to AAG concentrations [15].

A similar effect was observed with a formulation change. Relative to the phase I drug product, the phase II drug product consisted of smaller particle size and exhibited faster dissolution. Notably, the exposure to vismodegib was approximately threefold higher after a single dose with the phase II drug product (i.e., enhanced absorption, plasma AAG not saturated with a single dose), but the steady-state exposure was similar between the two formulations (i.e., due to saturation of AAG binding in plasma with continuous daily dosing) [4]. When a single dose of vismodegib is co-administered with rabeprazole, a 42 % decrease in mean AUC_0–24h_ is observed, as plasma AAG is not saturated and absorption is higher compared with steady state. However, after multiple doses of vismodegib, a 14 % decrease in AUC_0–24h_ is observed, as AAG is saturated and absorption is reduced. Each of these cases illustrates that vismodegib exposure may be influenced by absorption when AAG binding is not saturated in plasma (under single-dose conditions), but with continuous dosing, steady-state vismodegib exposure is highly influenced by levels of AAG in plasma. In addition, it had been shown previously that the absolute bioavailability of vismodegib was less than 10 % at steady state; thus, it seemed unlikely that alteration of gastric pH would decrease this parameter much further [3].

Results of an in vitro study in MDCK cells overexpressing P-gp suggest that vismodegib is a weak substrate of P-gp. However, considering that these were P-gp-overexpressing cells and the digoxin efflux ratio (ER) for vismodegib was approximately 1/10th that of the positive control (ER of 8.6 vs. 72.0, respectively), vismodegib did not appear to be efficiently transported by P-gp [14]. In addition, the ER of vismodegib was assessed in Caco-2 cells. In Caco-2 cells, the ER was 0.79, which supported the hypothesis that vismodegib was not a good substrate of P-gp. These data are not surprising because vismodegib has high permeability in Caco-2 cells [16]. Considering that vismodegib is inefficiently transported by P-gp, the contributions of AAG and solubility-limited absorption to the systemic exposure of vismodegib would be expected to far outweigh any potential impact of P-gp inhibition. Indeed, on day 1 the exposure of vismodegib was slightly lower when co-administered with itraconazole, which is the opposite effect of what should be observed if there were a P-gp interaction. As expected, co-administration of vismodegib with itraconazole (a strong inhibitor of both P-gp and CYP3A4) had no effect on total and unbound vismodegib *C*_ss_.

Vismodegib elimination involves multiple pathways but is primarily metabolized by CYP2C9 and CYP3A4, with several minor metabolites being produced by other CYP enzymes [14]. Notably, in vitro metabolic stability data suggest that the molecule is not metabolized efficiently, with 96 % of parent remaining after a 3-h incubation with human hepatocytes. Clinical observations show that vismodegib exhibits slow clearance following an intravenous dose, and parent compound is the predominant circulating drug species in plasma [6, 7]. Given the slow elimination of vismodegib via multiple elimination pathways, including metabolism by several CYPs and excretion of unchanged drug, clinically meaningful CYP-mediated drug interactions in which vismodegib is a victim are unlikely. Co-administration of vismodegib with a strong CYP3A4 inhibitor, itraconazole, had no impact on vismodegib *C*_ss_, and co-administration of vismodegib with fluconazole, a moderate inhibitor of CYP2C9, resulted in increases of approximately 31 % in mean AUC_0–24h_ and *C*_ss_,_ave_ values at steady state. *C*_ss_,_ave_,_u_ was approximately 1.57-fold higher following co-administration with fluconazole, which suggests modest inhibition of CYP2C9-mediated metabolism of vismodegib by fluconazole. However, these changes are not deemed to be clinically meaningful as vismodegib is not a narrow therapeutic index medication, and there was no evidence for an increase in AEs when vismodegib was combined with fluconazole compared with administration of vismodegib alone. Furthermore, the CYP2C9 genotype was looked at retrospectively, though no poor CYP2C9 metabolizers were recruited. Exposure of vismodegib was generally similar for intermediate and extensive metabolizers within each treatment arm, indicating that the CYP2C9 genotype did not appear to influence the steady-state PK of vismodegib in this study.

Co-administration of vismodegib with rabeprazole, itraconazole, or fluconazole was well tolerated. All the AEs were mild in severity and had resolved by the end of the study. The most frequently reported AEs (headache, constipation, nausea, and diarrhea) occurred with a similar frequency across all treatment arms.

In conclusion, the results from this clinical study in healthy volunteers demonstrated no clinically significant PK interaction between vismodegib and rabeprazole, itraconazole, and fluconazole. Therefore, clinically significant PK interactions between vismodegib and pH-elevating agents, P-gp inhibitors, and CYP450 inhibitors are not expected in the clinic. Results from this study will be used to update the vismodegib label worldwide.

## Electronic supplementary material

Below is the link to the electronic supplementary material.

Supplementary material 1 (PDF 51 kb)
