# Supplementary material for: A clinical drug–drug interaction study to evaluate the effect of a proton-pump inhibitor, a combined P-glycoprotein/cytochrome 450 enzyme (CYP)3A4 inhibitor, and a CYP2C9 inhibitor on the pharmacokinetics of vismodegib
Source: Cancer Chemother Pharmacol. 2016 May 6;78:41–9. doi: 10.1007/s00280-016-3020-z (PMC4921109; doi:10.1007/s00280-016-3020-z)
Supplement: Supplementary file 1 — Supplementary material 1 (PDF 51 kb) [file 280_2016_3020_MOESM1_ESM.pdf]

Supplemental Table 1 Baseline demographics

	Arm A Vismodegib (*n* = 22)	Arm B Vismodegib + rabeprazole (*n* = 24)	Arm C Vismodegib + itraconazole (*n* = 22)	Arm D Vismodegib + fluconazole (*n* = 24)	Overall (*N* = 92)
Age, mean (min, max), years	55 (36, 68)	59 (43, 70)	55 (41, 68)	57 (38, 68)	57 (36, 70)
BMI, mean (min, max), kg/m^2	28 (21, 34)	26 (20, 32)	28 (20, 34)	28 (22, 33)	27 (20, 34)
Sex, n (%)					
Female	22 (100)	24 (100)	22 (100)	24 (100)	92 (100)
Race, n (%)					
Asian	0	0	0	1 (4.2)	1 (1.1)
Black or African American	4 (18.2)	5 (20.8)	7 (31.8)	7 (29.2)	23 (25.0)
Multiple[a]	1 (4.5)	0	0	0	1 (1.1)
White	17 (77.3)	19 (79.2)	15 (68.2)	16 (66.7)	67 (72.8)
Ethnicity, n (%)					
Hispanic or Latino	7 (31.8)	6 (25.0)	7 (31.8)	7 (29.2)	27 (29.3)
Not Hispanic or Latino	15 (68.2)	18 (75.0)	15 (68.2)	17 (70.8)	65 (70.7)
AAG, geometric mean (CV%)					
Low (0.5–0.73 g/L)	5 (22.7)	5 (20.8)	5 (22.7)	6 (25.0)	21 (22.8)
Medium (0.74–0.96 g/L)	10 (45.5)	12 (50.0)	10 (45.5)	11 (45.8)	43 (46.7)
High (0.97–1.2 g/L)	7 (31.8)	7 (29.2)	7 (31.8)	7 (29.2)	28 (30.4)

AAG alpha-1-acid glycoprotein, *BMI* body mass index, *CV%* coefficient of variation

[a] Multiple = White and American Indian or Alaska Native

Supplemental Table 2 Treatment-emergent AEs with incidence of ≥4 patients in the overall population

AE (preferred term)	Arm A Vismodegib (*n* = 22)	Arm B Vismodegib + rabeprazole (*n* = 24)	Arm C Vismodegib + itraconazole (*n* = 22)	Arm D Vismodegib + fluconazole (*n* = 24)	Overall (*N* = 92)
Headache	4 (18.2)	3 (12.5)	2 (9.1)	3 (12.5)	12 (13.0)
Constipation	4 (18.2)	6 (25.0)	0	1 (4.2)	11 (12.0)
Nausea	2 (9.1)	0	3 (13.6)	4 (16.7)	9 (9.8)
Diarrhea	1 (4.5)	2 (8.3)	4 (18.2)	1 (4.2)	8 (8.7)
Abdominal pain	1 (4.5)	1 (4.2)	3 (13.6)	2 (8.3)	7 (7.6)
Pruritus	0	2 (8.3)	3 (13.6)	1 (4.2)	6 (6.5)
Abdominal distention	1 (4.5)	4 (16.7)	0	0	5 (5.4)
Flatulence	1 (4.5)	2 (8.3)	1 (4.5)	0	4 (4.3)
Dizziness	2 (9.1)	0	2 (9.1)	0	4 (4.3)
Rash	0	0	3 (13.6)	1 (4.2)	4 (4.3)
